# Understanding the barriers and facilitators related to birthing pool use from organisational and multi-professional perspectives: a mixed-methods systematic review

**DOI:** 10.1186/s12978-023-01690-0

**Published:** 2023-10-04

**Authors:** Megan Cooper, Anna-Marie Madeley, Ethel Burns, Claire Feeley

**Affiliations:** 1 Flinders University, Adelaide, Australia; 2 Open University, Milton Keynes, England; 3 Oxford Brookes University, Oxford, England; 4 King’s College London, London, England

**Keywords:** Analgesia, Birth pool, Childbirth, Guidelines, Maternity care, Midwifery, Physiological birth, Policies, Water immersion, Water birth, Obstetrics, Anesthesiology, Neonatology

## Abstract

**Aims:**

To identify and synthesize the evidence regarding the facilitators and barriers relating to birthing pool use from organizational and multi-professional perspectives.

**Design:**

A systematic integrated mixed methods review was conducted.

**Data sources:**

MEDLINE, CINAHL, PsychINFO, EMCARE, PROQUEST and Web of Science databases were searched in April 2021, March 2022 and April 2024. We cross-referenced with Google Scholar and undertook reference list searches.

**Review methods:**

Data were extracted from studies meeting the inclusion criteria. Barriers and facilitators to birthing pool use were mapped and integrated into descriptive statements further synthesized to develop overarching themes.

**Results:**

Thirty seven articles (29 studies) were included—quantitative (12), qualitative (8), mixed methods (7), and audits (2), from 12 countries. These included the views of 9,082 multi-professionals (midwives, nurses, obstetricians, neonatologists, students, physicians, maternity support workers, doulas and childbirth educators). Additionally, 285 institutional policies or guidelines were included over 9 papers and 1 economic evaluation. Five themes were generated: *The paradox of prescriptiveness, The experienced but elusive practitioner, Advocacy and tensions, Trust or Trepidation* and *It’s your choice, but only if it is a choice.* These revealed when personal, contextual, and infrastructural factors were aligned and directed towards the support of birth pool use, birthing pool use was a genuine option. Conversely, the more barriers that women and midwives experienced, the less likely it was a viable option, reducing choice and access to safe analgesia.

**Conclusion:**

The findings demonstrated a paradoxical reality of water immersion with each of the five themes detailing how the “swing” within these factors directly affected whether birthing pool use was facilitated or inhibited.

**Supplementary Information:**

The online version contains supplementary material available at 10.1186/s12978-023-01690-0.

## Abstract (plain language summary)

During childbirth, most women wish to use a pain management technique; some prefer to use medications and others prefer non-medication methods. Another option is to use a birthing pool, larger than a typical bath, that encourages buoyancy and the freedom to move. The warm water can also offer comfort, relaxation and pain relief. Extensive studies have demonstrated birthing pool use during childbirth is safe for mothers and babies. It is associated with reduced medical interventions (e.g. speeding up labour and cuts to the perineum), improved outcomes (e.g. satisfaction, less pain or excessive bleeding after birth). Given these positive outcomes, we wanted to explore any barriers or facilitators for birthing women and people accessing birthing pools so we could help improve access for those wishing to use one. We gathered and assessed the literature to explore these aspects. We included quantitative and qualitative studies exploring the perspectives of different maternity professionals and those of organisations. We reviewed 37 studies from 12 countries (from 2004 to 2020). We found that birthing pool use was a viable option when all maternity professional groups and their organisational guidelines or policies valued and supported its use. Conversely, in other organisations, multiple barriers prevented the use of birthing pools as an equally viable option to medication pain relief options. These barriers were influenced by the beliefs of different maternity professional groups, organisational guidelines or policies (where some were highly restrictive) whether midwives were supported to offer care in birthing pools or whether the midwives had the confidence to do so.

**Supplementary Information:**

The online version contains supplementary material available at 10.1186/s12978-023-01690-0.

## Background

The pain of childbirth is unique. It is both expected and purposeful, unlike other forms of pain. Many women seek interventions to ease the discomfort. While some will opt for pharmacological methods that attempt to eliminate pain, such as epidural analgesia, other women will choose non-pharmacological options that buffer the pain and may increase the likelihood of a physiological birth [1]. Water immersion for labour and/or birth in an appropriately sized birthing pool, is a low-tech, non-pharmacological pain relief option. As an effective tool to optimize the physiology of labour and the release of endogenous endorphins and oxytocin [2], water immersion is associated with several benefits: reduced labour duration [3], transfer from midwifery-led settings [4], episiotomy, postpartum hemorrhage, [5] and carries no increased risk of obstetric anal sphincter injury (OASI) [5]. Furthermore, birth pool use is associated with an increased likelihood of spontaneous vaginal births [6], intact perineum [7] and high maternal satisfaction [8]. Importantly it can reduce intervention in the obstetric unit setting [3, 5, 9], particularly for nulliparae.

For neonates, systematic reviews have not found an association with waterbirth and poor neonate outcomes [5, 10]. While more cord avulsions have been reported for waterbirths, the incidence is widely variable indicating care practices such as undue traction may be more relevant than birth in water [5]. Moreover, cord avulsion can be quickly and easily managed with no consequences for the newborn [10]. Despite these benefits and with no long-term adverse outcomes for neonates—as identified in multiple systematic reviews [10–12], barriers to birthing pool use remain [13–15]. There have been calls for a large-scale randomised controlled trial (RCT) to determine the safety profile. However, as an intervention, the birth pool precludes blinding, which presents a major RCT limitation and a recent feasibility study (N = 1260) found only 15% (n = 118) of participants would consent to randomisation in a future waterbirth trial, indicating that an RCT is unlikely to be feasible [16], or ethical in a healthcare climate that respects and supports maternal choice. Therefore, an understanding of these barriers, along with potential facilitators for birthing pool use is important for care providers and maternity services to ensure equitable and improved access.

With all the benefits water immersion offers birthing women and people, demand for birth pool access and use has increased. Use in high-income countries has grown steadily since the 1990s [12], prompting the development of numerous policies and guidelines regarding birthing pool use [17, 18]. However, evidence from the UK and Australia suggests that birth pool usage is dependent upon the setting, staffing and infrastructure [17, 19, 20]. For example, the UK Birthplace study found that comparable healthy women planning to birth in obstetric units (OU) were significantly less likely to use water immersion compared to women who birth in midwifery-led settings [21]. For example, only 13.3% of nulliparae used immersion birthing pool in an OU versus 53.7% in a freestanding midwifery unit (FMU), with a similar disparity amongst multiparae [21].

In other high incomes countries, uptake of water immersion also varies by setting [22]. In Australia, there is a significant disparity across States and territories and within and across local health networks with the highest birth pool use in midwifery-led settings and continuity of care models. It is estimated that around 60–80% of women birthing at home in Australia use water immersion for labour and/or birth compared with rates between 1 and 10% in hospital settings [23, 24]. These statistics highlight the known association between birthing pool use and midwifery-led care and settings [6]; suggesting birth pool access may be inhibited/less supported in the obstetric unit (OU) setting [21]. Given that the majority of birthing women and people in high-income countries birth in OU’s [25], where they are cared for by multi-professional teams (midwives, obstetricians, anaesthetists, and paediatricians), exploration of the perspectives from all maternity professionals and a review of organisational evidence (i.e. policy and/or guideline analysis) would yield a comprehensive overview of barriers and facilitators regarding birth pool use. As such, this mixed methods evidence synthesis, the first to date was undertaken to examine the organisational and multi-professional perspectives of water immersion to uncover both the facilitators and barriers to birth pool use.

## Methods

### Aim

The aim of this mixed-methods evidence synthesis was to source, synthesize and interpret the evidence relating to birthing pool use from organisational (policies and guidelines analyses) and multi-professional perspectives to examine the barriers and facilitators to birth pool use.

### Design

We used a systematic integrated mixed-methods design. This deviated from the original published protocol (PROSPERO 2019 CRD42019146998) whereby we originally intended to carry out a qualitative meta-thematic synthesis exploring maternity multi-professionals’ views and experiences regarding birthing pool use during labour and birth. Our initial scoping searches identified less qualitative studies than anticipated but did yield surveys and papers related to organizational perspectives of birthing pool use. Therefore, we expanded our research questions and amended our research design and search strategy to secure a fuller, more comprehensive review to answer our research questions. We adopted a mixed methods integrated review design, as per Noyes, Booth, Moore, Flemming, Tunçalp and Shakibazadeh [26]. This involved using both quantitative and qualitative data which were gathered, analysed and integrated to answer the research question: *‘What are the barriers and facilitators, from organisational and multi-professional perspectives, for birthing pool use for labour and/or birth?’.*

### Reflexivity

Reflexivity is an integral part of quality research; researchers convey their positioning in relation to the research to enhance the trustworthiness of the study [27]. In brief all authors are midwives. CF, AM and EB have extensive experience of facilitating water immersion for labour and birth across all birth settings (home, birth centre, and hospital), and MC has extensive experience of facilitating birth pool use in the OU setting. All believe water immersion is a feasible low-cost, low-tech form of pain relief with benefits for women. Additionally, all believe access to water immersion needs to be improved to ensure equitable pain relief options are available. By referring back to or reflecting on our prior positioning throughout the research process, potential blind spots or biases were challenged [27]. Furthermore, three authors have been involved in water immersion research in various capacities with MC directly involved in 5 of the included papers in this review. Therefore, to ensure impartial assessment of quality, a fourth researcher AM reviewed these papers who was unknown to MC at the time of assessment, with CF.

### Search strategy

We carried out two pilot searches in MEDLINE and Cumulative Index of Nursing and Allied Health Literature (CINAHL) and found that using a simplified search string yielded the best balance between comprehensive searching and specificity. See Table 1 for the final search terms. All searches can be found in Additional file 1.

**Table 1:** Search terms

midwife* or midwives or midwifery or obstetri* or nurs* or anaesth*or paediatric* or neonat*	
AND water birth or water?birth or birthing pool or under water birth or birthing pool or tub or hydrotherapy	

A second search was carried out using the identified key words in Cumulative Index of Nursing and Allied Health Literature (CINAHL), MEDLINE (Ovid), PsychInfo, Embase, Emcare, Proquest and Web of Science. These searches were then cross-checked with Google Scholar. Reference/citation checking of all identified papers was undertaken. Details of all search results are provided in Additional file 1. Database searches were saved to EndNote and duplicates then removed.

Inclusion and exclusion criteria were adapted to reflect the expanded review. Accordingly, we focused on the views, opinions, perspectives and experiences of all maternity staff (midwives, obstetricians, anesthetists, neonatologists) regarding birthing pool use. Additionally, we sought policy and/or guideline analyses, economic evaluations or audits to reflect organizational perspectives. It is also important to note differences between countries in their use of clinical policies and/or guidelines. For example, in the UK birthing pool use sits within guidelines—flexible recommendations for care provision. Whereas in Australia, birthing pool use sits within policies (mandatory expectations of clinical care provision) *and* guidelines (flexible recommendations of care). While we recognise these differences, in reality, guidelines are often viewed as mandatory policies [28] so for the purposes of this review we have kept them closely aligned and representative of organisational perspectives. We also included any published study, thesis or audits related to the research questions e.g., quantitative, qualitative, mixed-methods or policy/guideline analysis. No date restrictions were set. No language caveat other than studies that could be translated via Google Translate. No time restrictions were applied but papers were excluded if they could not be translated and if they related to either the views and experiences of women using birthing pools or those related to maternal-neonatal outcomes following birthing pool use.

### Quality appraisal

Quality assessment of cross-sectional surveys was carried out using a critical appraisal checklist [29]. Quality assessment of qualitative papers was carried out using criteria from Walsh and Downe [30]. Quality assessment of economic evaluations were carried out using the adapted CASP tool by Drummond, Sculpher, Claxton, Stoddart and Torrance [31]. Quality assessment of mixed methods papers was carried out using the Mixed Methods Appraisal Tool (MMAT) tool [32] where they reported both elements of the mixed methods in one paper. However, for mixed method studies reporting discrete phases across different papers, they were quality assessed against the framework suited to the research design identified in the paper and not with the MMAT tool. For example, Russell [20] and Russell, Walsh, Scott and McIntosh [33] were publications arising from one overarching mixed methods action research study. Russell [20] reported the qualitative component comprising of interview and focus group data and was quality assessed against the Walsh and Downe [30] framework. Russell, Walsh, Scott and McIntosh [33] reported the distinct quantitative component comprising of a survey following an educational workshop and was quality assessed against the BMJ Publishing Group Ltd. [29] survey quality framework. Audits were not quality assessed but all other studies were graded A-D by discussion between two reviewers (CF/AM), where A: No, or few flaws; the study credibility, transferability, dependability, and confirmability is high, and D: Significant flaws that are very likely to affect the credibility, transferability, dependability, and/or confirmability of the study [30]. A detailed exposition of the quality assessments can be found in Additional file 2.

### Data analysis and synthesis

Data analysis was undertaken in stages (an exposition of the full analysis found in Additional file 1). First, study characteristics of the included papers were collected on a data extraction form: author and date, title, resource setting, country, study design, setting, population, participants, methods. Second, a data-based convergent synthesis as per Noyes, Booth, Moore, Flemming, Tunçalp and Shakibazadeh [26] was carried out. This involved MC reading and re-reading each text ‘line by line’ and extracting the findings from the papers in short relevant sections that were tabulated, colour coded and applied with a code word or phrase. Where studies also included women’s views, data only pertaining to staff views was extracted. CF cross-checked at this stage of the process to ensure accuracy and trustworthiness of the analysis. Third, the codes were then reviewed and grouped together which generated 10 descriptive categories reflecting five sets of opposing statements related to the key barriers and facilitators to birthing pool use. Finally, these opposing statements were further synthesized interpretatively into five themes, reviewed by the whole research team.

## Results

**Fig. 1:**
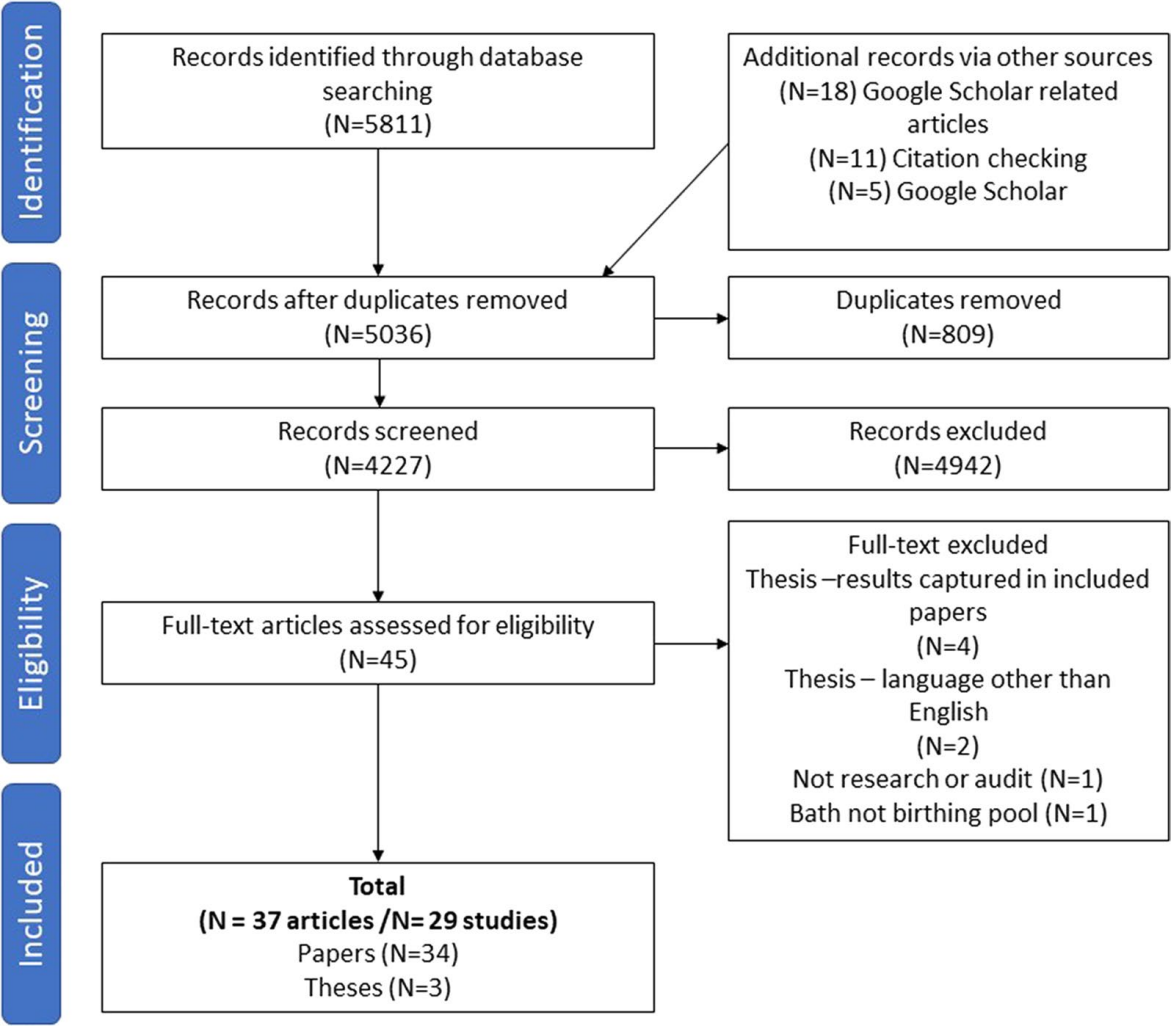
PRISMA flow diagram

The initial literature search was undertaken in April 2021 by MC and updated in March 2022. CF carried a further search update April 2023 (no further studies were found). A total of 5788 records were sourced from the databases and through Google Scholar and citation checking (see Fig. 1 for PRISMA flow diagram). Duplicates were removed. Paper titles and abstracts were screened against the inclusion criteria by MC and irrelevant records were excluded yielding 93 records in total. Full text versions of 11 papers could not be sourced therefore, were excluded prior to full-text screening. Thereafter, full text screening of 82 records was performed separately by MC and CF and a consensus reached about each paper yielding a total of 44 papers that met the inclusion criteria. However, of these, seven records were later excluded as the results of four theses were included as published papers (already included), while another two theses were excluded as they were not written in English and were too large for Google Translate, and finally, one was excluded as it was not research or audit. Therefore, the total sample included was 37 papers, but 29 studies—5 authors utilized either mixed-methods or reported on different aspects of the same overarching study [13, 14, 17, 19, 33–41].

### Study characteristics

The characteristics of each of the included studies can be found in Table 2. Included papers were primarily from high income countries: Australia (16), UK (6), USA (5), Canada (2), New Zealand (1), Scotland (1), Spain (1), Italy (1), Sweden (1), Japan (1) and France (1), with one paper, in India, a lower-middle income country. The overarching research designs of the included 29 studies were—quantitative (12), qualitative (8), mixed methods (7), and audits (2). These included the views and experiences of 9,082 maternity professionals: midwives (5,633), nurse-midwives (n = 1684), nurses (836), student midwives (356), obstetricians (400), pediatrician/neonatologists (47), physicians (75), maternity support workers (9), doulas (40) and childbirth educators (2). Additionally, a total of 285 institutional policies or guidelines were included over 9 papers and 1 economic evaluation.

**Table 2:** Study characteristics

	Author, date, country and setting	Aim (s)	Design/theoretical perspective and/or methodology	Sample selection method	Sample size and characteristics	Data collection and analysis	Quality grade
1	Almoghrabi, H, 2018 US online	To generate scientific knowledge regarding the US CNM’s/ CM’s and student midwife’s Knowledge, Attitude, and Practice (KAP) of water birth (WB)	Quantitative Theory of Planned Behavior (TPB) Quantitative descriptive survey	Purposive Eligibility—ACNM membership—CM’s (Certified Midwives), CNM (Certified Nurse Midwives), and midwifery Students	764 midwives, 197 midwifery students	Survey Descriptive statistics of central tendency, frequencies and percentages Open-ended questions, thematic analysis	B
2	Baba, K., et al., 2016 Japan Urbanized Kanto region of Japan, which includes Tokyo, Kanagawa, Saitama and Chiba	To survey the policy implementation regarding care during the second stage of labor at Japanese hospitals, clinics, and midwifery birth centers, and to compare those policies with the recommendations in guidelines for midwives	Quantitative Cross-sectional survey	Purposive Eligibility—institutions with an obstetric ward, clinics and midwifery birth centers	255 maternity institutions 118 hospitals, 66 clinics, 71 midwifery birth centers	Survey Statistical quantities and frequency distributions Chi-square and Fisher’s exact test < 0.05 statistically significant	C
3	Barasinski, C., et al., 2018 France	To describe the practices reported by French midwives during labor (first stage and passive phase of the second stage)	Quantitative Cross-sectional internet questionnaire	Purposive Eligibility—Midwives who attended at least one childbirth in 2013 and who performed deliveries in equipped facilities legally required for providing deliveries (i.e., not home deliveries) were eligible	1496 midwives (from 377 maternity units)	Internet questionnaire Chi-square or Fisher’s exact test to compare qualitative variables. Student T for quantitative variables P < 0.05	B
4	Baxter L., 2006 UK One maternity unit	To share audits comparing ‘pool users’ with ‘pool births’ and reflects on the experience of the midwives	Audit	Purposive Eligibility—Midwives working at the unit	Unreported	Clinical audit and discussion following implementation of installed birthing pools	N/A
5	Bayes, S., et al., 2019 Australia	To investigate midwives’ experiences of leading practice change	Qualitative Glaserian Grounded Theory methodology	Purposive sampling Eligibility—Midwives who had led practice change initiatives	16 Australian midwife change leaders	Single in-depth semi-structured interview conducted in person Grounded theory techniques	A/B
6	Carolan-Olah, M., et al., 2015 Australia One maternity unit	To explore midwives' experiences and views of the factors that facilitate or impede normal birth in a hospital setting	Qualitative Interpretative Phenomenological approach (IPA)	Purposive sampling Eligibility—Registered Midwives working on the birthing suite	22 midwives	In-depth interviews approximately one hour Smith and Osborn's (2008) IPA approach	A/B
7	Chapman, B., 2004 New Zealand (North Island only)	Review of protocols for waterbirth obtained from five North Island hospitals in New Zealand compared against available literature	Qualitative Evaluation and comparison of hospital protocols against literature findings	Purposive Eligibility- all maternity hospitals in the North Island	5/ 17 hospital protocols were received and included	North Island (NZ) hospital water immersion protocols Common elements were grouped into the following major elements	B/C
8	Cooper, M., McCutcheon, H., and Warland, J., 2017^a^ Australia	To determine how water immersion policies and/or guidelines are informed, who interprets the evidence to inform policies/guidelines and to what extent the policy/ guideline facilitates the option for labour and birth	Mixed methods (convergent parallel design) Phase 1 qualitative component Critical theory and critical discourse analysis	Purposive Eligibility—All maternity hospitals in Australia	25 WI for labour and/or birth policies were sourced - eight were policies and 17 were guidelines	Hospital policies and guidelines Parker’s ten criteria and Fairclough’s three-dimensional model for analysis of text, discourse and society	A/B
9	Cooper, M., McCutcheon, H., and Warland, J., 2019^a^ Australia	To determine how water immersion policies and/or guidelines are informed and to what extent the policy/guideline facilitates the option of water immersion for labour and birth with respect to women’s choice and autonomy	Mixed methods (convergent parallel design) Phase 2 Qualitative component Critical, post-structural interpretive interactionism	Purposive Eligibility—Individuals who had been involved in the development of policies and /or guidelines for WI during labour and/or birth	12 participants, 11 midwives, 1 obstetrician	Semi-structured, open-ended interviews Seven steps of critical, post-structural interpretive interactionism	A
10	Cooper, M., McCutcheon, H., and Warland, J., 2020^a^ Australia	Determining the extent to which midwives felt they were able facilitate WI and more, their views of women’s choice and autonomy with respect to the option	Mixed methods (convergent parallel design) Phase 3 Quantitative component Critical, post-structural interpretive interactionism Survey	Purposive and snowballing Eligibility—Midwives who were members of Australian College of Midwives (ACM)	233 midwives	Online survey adapted from Meyer et al., 2010 including open text responses Steps 4–7 of critical, post-structural interpretive interactionism	A
11	Cooper, M., Warland, J., and McCutcheon, H., 2018^a^ Australia	To explore midwives’ knowledge, experiences and support for the option of water immersion for labour and birth in practice and their involvement, if any, in development of policy and guidelines pertaining to the option	Mixed methods (convergent parallel design) Phase 3 (part 2) Quantitative component Web-based survey	Purposive and snowballing Midwives who were members of Australian College of Midwives (ACM) (distribution of e-bulletin)	234 midwives	Online survey Statistical and visual analysis through SPSS 20. Descriptive statistics %, mean and median. Chi-square tests with monte-carlo exact and Friedman test	A/B
12	Cooper, M., Warland, J., and McCutcheon, H., 2019^a^ Australia	To examine how accreditation requirements are reflected in policy and guideline documents, how those that were involved in the process of develop and implementation viewed the need for additional training and finally, how midwives’ viewed accreditation requirements with respect to their ability to uphold women’s autonomy and choice surrounding the practice	Mixed methods (convergent parallel design) Phase 3 (part 3) Qualitative component Critical, poststructural interpretive interactionism	Purposive and snowball sampling Eligibility— P1 And/all Australian WI policies or guidelines in P 2 and 3: Practicing maternity staff	P1: Australian policies and guidelines pertaining to the use of water P2: 12 participants—11 midwives, 1 obstetrician P3: 234 Australian midwives	P1: Policy documents P2: Semi-structured open-ended interviews P3: Online survey P1: Critical discourse analysis P2: Critical, poststructural interpretive interactionism P3: Statistical and thematic analysis	A
13	Freeman, L. M. and K. Griew, 2007 Australia	To show that the allocation of an active voice to the woman within practice guidelines serves the interest of all parties within the health care relationship. To illustrate women’s involvement in decision making within a clinical practice guideline the ‘Use of the Bath in Labour and Birth’	Qualitative Shared decision-making conceptual framework	Purposive Eligibility- Clinical practice guidelines in Australia and New Zealand	Unclear	Policy/guideline documents Comparison of policy against shared decision-making conceptual framework	C/D
14	Hammond, A., et al., 2014 Australia One birth centre and two labour wards	To explore the impacts of physical and aesthetic design of hospital birth rooms on midwives	Qualitative Video ethnographic study, thematic analysis of midwives interviews	Purposive and convenience sampling Eligibility – women and staff working within the hospitals who consented to be filmed	7 midwives, 1 midwifery student (6 women, 6 birth partners)	Individual video-reflexive, unstructured interviews Thematic analysis	B
15	Jessiman, W. C. and H. Bryers, 2000 Scotland One maternity unit	To examine the professional and educational issues surrounding the installation of the facility (water in labour) in Inverness, and the findings of an audit of the first two years of its use	Audit	Purposive Eligibility—Midwives working at the maternity unit	58 midwives	Audit tool was adapted from Burns and Greenish (1993) was gathered Frequency against Likert scale Words used to describe water immersion	N/A
16	Lewis L., et al., 2018 Australia Tertiary public maternity hospital with AMU	To examine midwives' education, knowledge and practice around immersion in water for labour or birth	Mixed methods A cross-sectional design: survey/qualitative descriptive	Purposive Eligibility—Midwives working in either Midwifery Group Practice (MGP) and Community Midwifery Program (CMP) who facilitated water immersion in the unit	Phase 1- 29 midwives Phase 2 –12 midwives (two focus groups)	Phase 1—Questionnaire Phase 2—45 min focus group Phase 1—means, interquartile ranges for continuous data, frequency for categorical data via SPSS, scenarios scored by four researchers Phase 2—Thematic analysis of transcribed focus groups	B
16	Madden, K. L., et al., 2013 Australia Large tertiary referral centre and country wide for survey	To compare the personal preferences of pregnant women, midwives and obstetricians regarding a range of physical, psychosocial and pharmacological methods of pain relief for childbirth	Quantitative Self-completed questionnaires	Purposive Eligibility—Obstetricians publicly listed as practicing obstetrics on the Royal Australian and New Zealand College of Obstetricians and Gynaecologists Practicing midwives at research sites (400 women booked into the hospital)	242 obstetricians 210 midwives (123 women)	Questionnaires Proportions were used to describe categorical data, means for continuous data and medians for ordinal data Participants’ preferences for pain relief methods used non-parametric tests, Kruskal–Wallis (H) and Mann–Whitney (U) to compare between groups using the Monte-Carlo method Findings statistically significant at p < 0.05	B
18	Mercredi, A., 2020 (THESIS) Canada Acute care facility (hospitals	To understand the level of support for waterbirth and explore the overall perceptions and experiences of childbirth HCPs in terms of perceived benefits, risks and barriers	Quantitative Nonexperimental, cross-sectional online survey	Purposive and snowballing sampling Eligibility – Currently practicing maternity professionals involved with childbirth care	214 registered nurses, 38 registered midwives, 41 physicians, and 11 obstetricians	Survey Descriptive statistics	B
19	Meyer, S. L., Weible, C. M., and Woeber, K., 2010 US Georgia statewide	To examine the perceptions, exposure to, and experience of a sample of Georgia CNMs with Waterbirth	Quantitative Survey questionnaire	Purposive Eligibility—Currently or recently active CNMs in Georgia	119 Certified Nurse-Midwives	Survey Descriptive statistics	C
20	Milosevic, S., et al., 2020^a^ UK Three obstetric units and three midwifery units	To identify factors influencing pool use through qualitative case studies of three obstetric units and three midwifery units in the UK	Mixed methods cohort study Phase 2 and 3 Qualitative component Case studies	Purposive sampling Eligibility—Women and maternity staff working at the study sites	111 participants including 57 midwives, 12 student midwives, 8 obstetricians, 4 pediatrician/ neonatologist, 7 midwifery support workers, and 2 doulas (21 postnatal women)	Semi-structured interviews, collation of service documents and public-facing information, and observations of the unit environment Deductive thematic analysis and systematic coding	A
21	Milosevic, S., et al., 2019^a^ UK	To identify factors influencing the use of birth pools	Mixed methods cohort study Phase 2 and 3 Qualitative component Descriptive	Opportunistic Eligibility—unclear	21 midwives, 8 obstetricians, 6 paed/neonatologist (85 women)	Online discussion groups and semi-structured interviews Thematic analysis	B
22	Muñoz-Sellés, E., et al., 2013 Spain 28 hospitals in Catalonia, Spain accredited as public normal birth centres	To describe the professional profile of midwives who provide care for natural childbirth in Catalan hospitals accredited as centres for normal birth, to assess midwives’ levels of training in CAT and their use of these therapies and to identify specific resources for CAT in labour wards	Quantitative Descriptive cross-sectional survey	Purposive Eligibility- Qualified midwives who had worked for at least 6 months in one of the hospitals in Catalan	237 midwives	Questionnaire based on Sara and Hastings-Tolsma (2009) Descriptive statistics—frequencies for categorical variables, means, standard deviations, medians, minimums, maximums Chi-square and student t-test for comparisons Pearson’s correlation p < 0.05 significant	B/C
23	Newnham, E. C., McKellar, L. V., and Pincombe, J. I., 2015^a^ Australia Metro tertiary hospital labour ward	To examine personal, social, institutional and cultural influence on women in their decision to use epidural in labour and a comparison of policy and information pamphlets for using epidural or water in labour	Qualitative Critical medical anthropology (CMA) Ethnography	Purposive Eligibility—Maternity staff and women who consented to be observed Documentation included client information handouts, hospital policies, state department policy	Maternity staff present at the time of observations (unclear numbers) (16 women/ 6 women consented to presence of researcher at birth) Documentary review	Participant observation and informal interviews with staff Detailed noted of observations, conversations and interactions CMA framework Document analysis	A
24	Newnham, E., McKellar, L., and Pincombe, J., 2017^a^ Australia Metro tertiary hospital labour ward	To investigate personal, social, institutional and cultural influences on women making decisions about using epidural analgesia in labour which we juxtapose with similar processes relating to use of water for labour and/or birth	Qualitative Ethnography Critical medical anthropology (CMA)	Purposive Eligibility—Maternity staff and women who consented to be observed	Maternity staff (unclear numbers) (16 women)	Participatory observation Sequential interviews Field notes CMA framework	A
25	Nicholls, S., et al., 2016 Australia Four public maternity services in a metropolitan area	To capture midwives’ perceptions of becoming and being confident in conducting waterbirth	Qualitative Grounded theory	Purposive sampling Eligibility—Qualified midwives working at the four public hospitals in WA	26 midwives	One-to-one interviews Focus groups Thematic analysis	A/B
26	Orrantia, E. and C. Petrick, 2020 Canada Northern Ontario state wide	To understand the beliefs and perspectives of women in northern Ontario and their obstetrical providers with respect to water birthing as access to this service is limited in this region	Quantitative Survey	Opportunistic Eligibility- Women of childbearing age and maternity professionals from four locations in northern Ontario	33 midwives 56 registered nurses 34 family physicians 11 obstetricians (400 women)	Two surveys – patient and health care professionals Proportions and 95% confidence intervals (CIs) were calculated for each of the response options Chi-square tests, t tests, p < 0.05	C/D
27	Pagano, E., et al., 2010 Italy One hospital	To assess the cost-effectiveness of water compared with normal delivery	Quantitative Economic evaluation from retrospective cohort study	Matched cohort study Eligibility—All nulliparous women who had birthed within the time parameters	(110 women who had a waterbirth, 110 who had a land birth during the same period.)	Retrospective case note review Analysis comparing groups Economic evaluation	A/B
28	Plint, E., and Davis, D., 2016 Australia Tertiary level hospital with AMU	To describe and compare the attitudes and practices of midwives and obstetric doctors in a tertiary setting regarding water immersion for labour and birth and to identify strategies for improving bath usage in the facility	Mixed methods Survey	Purposive Eligibility—All employed midwives and obstetricians who provided labour care in the facility in the prior 12 months	13 obstetricians, 62 midwives (49 birth suite midwives, 13 continuity midwives)	Online and hard copy questionnaire adapted from Russell (2014) Mean and median scores were determined for each professional group, Mann–Whitney U using Fisher’s exact test or continuity correction Open-ended text analysed descriptively	A/B
29	Russell, K., 2011^a^ UK One hospital labour ward	Using action research to identifying inequalities in the availability of water birth on one hospital labour ward	Mixed methods Qualitative component of action research study Critical theory and critical discourse analysis	Purposive Eligibility—Midwives who regularly worked on labour ward at the study site	16 midwives	Face-to-face interviews Focus groups Structural and interactional analysis	A/B
30	Russell, K., et al., 2014^a^ UK One obstetric-led hospital	Using action research and follow up questionnaire to explore improving the availability of hydrotherapy and waterbirth in one UK labour ward	Mixed methods Quantitative component of action research study	Purposive Eligibility—Labour ward coordinators and midwives working at the study site	105 midwives (9 labour ward coordinators involved in workshop and 96 midwives completed questionnaires)	Problem solving workshop Survey based on Davies and Hodnett (2002) Tests for normality on the distribution of scores for Total Personal Knowledge, Total Waterbirth Self-efficacy and Total Social Support (Kolmogorov–Smirnov > 0.05, Histograms and Q–Q plots). One-way ANOVA with post-hoc Tukey tests	A/B
31	Seibold, C., et al., 2010 Australia Major metropolitan maternity hospital	To explore and describe midwives’ perceptions of birth space and clinical risk management and their impact on practice both before and after a move to a new facility	Qualitative Exploratory descriptive study with modified participatory approach and observation	Purposive Eligibility—midwives working at the study site	18 midwives including graduate, caseload and hospital midwives	Focus groups with three groups Field notes from birth space both before and after moving to new site Modified participatory and observation Framework analysis	B
32	Stark, M. A. and M. G. Miller, 2009^a^ US Country wide	To determine nurses’ perceived barriers to the use of hydrotherapy in labour	Quantitative Comparative descriptive survey design	Convenience and purposive Eligibility—Nurses attending a national convention and members of perinatal listserves who had provided care to a laboring woman in the last 12 months	401 intrapartum nurses	Online and paper-based survey (?revised from pilot below, not clear) Variance. Bonferroni’s post hoc analyses were performed .05 was determined a priori	A/B
33	Stark, M. A., and Miller, M. G., 2010^a^ US Country wide	To develop and test an instrument of nurses’ perceptions of the barriers to the use of hydrotherapy in labour	Quantitative Griepp’s (1992) Model of Ethical Decision Making in the Management of Clients’ Pain Scale/questionnaire development	Convenience and purposive Eligibility- Nurses attending a national convention and members of perinatal listserves who had provided care to a laboring woman in the last 12 months	65 intrapartum nurses	Phase I—Online survey (?pilot not clear) For each item, range, mean, standard deviation and distribution were examined Correlations were determined Construct validity against Labour Support Scale	A/B
34	Sushma, Y., et al., 2019 India One general hospital	To assess the level of knowledge of waterbirth among staff nurses	Quantitative Descriptive design Questionnaire	Convenience sampling technique Eligibility- Nurses at study site	100 nurses	Questionnaire Frequencies, percentages, mean, standard deviation, Chi square tests	D
35	Ulfsdottir, H., Saltvedt, S., and Georgsson, S., 2020 Sweden Country wide	To explore the experiences, knowledge and attitudes regarding waterbirth among midwives, obstetricians/ gynecologists and neonatologists	Mixed methods Cross-sectional survey	Purposive Eligibility—All maternity staff working within Swedish maternity wards	1467 midwives 105 obstetricians/gynaecologists 37 neonatologists	Mixed methods survey Descriptive statistics and quantitative content analyses Univariate comparisons between the professions were performed using Chi square, Fisher’s exact test and Mann Whitney U-test as appropriate. P-values < 0.05 were considered statistically significant Content analysis for qualitative data	B
36 US Country wide	Way, S. E., 2015 (THESIS)	To specifically examine perceived barriers to attending waterbirth as reported by CNM midwives	Quantitative The social amplification of risk framework Survey	Convenience sampling Eligibility—ACNM members	1,565 / 7,374 Nurse-Midwives	Online survey Descriptive statistics, frequency distributions, and nonparametric measures of correlation	C
37 UK Across all birthplace settings	Woodward, J. L., 2012 (THESIS)	To investigate the feasibility of a waterbirth RCT to assess the effects of a waterbirth on the neonate, to explore women’s thoughts about participation and whether randomisation affects women’s satisfaction with their childbirth experience and to assess midwives’ attitudes to waterbirths	Mixed methods Qualitative component (from wider feasibility study)	Opportunistic sample Eligibility—Practicing midwives at the time of the study	5 midwives (4 NHS, 1 Independent Midwife and worked bank shifts in local unit.)	Semi-structured interviews Thematic network analysis	C

^a^Same overarching study, different components published as individual papers

### Findings

The ten descriptive statements derived from the dataset can be found in Table 3.

**Table 3:** Descriptive statements

Review finding	Codes	Studies contributing to the findings
Education, training, mentorship, and experience leads to knowledge, competence, and confidence in facilitating water immersion	*Limited opportunities for education both during training and as midwives*	[1, 7, 11, 25, 34, 37]
	*Training and experience improved competence and competence*	[1, 4, 9–11, 15, 16, 19, 25, 26, 28, 32]
	*Additional training is not needed*	[12, 37]
	*Mentorship is key*	[1, 4, 25, 35, 37]
	*Midwifery-led spaces promote greater confidence*	[2, 16, 20, 21, 28, 30, 32]
Water immersion is a midwifery option in demand that facilitates physical and psychological benefits and normal physiological birth	*Facilitates normal birth*	[9, 16, 25, 35–37]
	*Reduces intervention and adverse events*	[9, 16, 19, 25, 37]
	*Promotes comfort, protection, relaxation, and a more positive birth experience*	[4, 9, 11, 16, 19, 20, 26, 37]
	*Decreased use of analgesia*	[4, 11, 19, 26, 28, 35, 37]
	*Promotes empowerment and control*	[16, 26, 37]
	*Demand*	[4, 11, 17, 21, 26]
	*Midwifery option*	[8, 26, 37]
Policies and guidelines can be facilitative and prompt implementation of water immersion	*Ensure safety for the woman and midwife*	[9, 11–13, 25, 37]
	*Alleviate practitioner concerns and promotes confidence*	[9, 25]
	*Improved accessibility and availability*	[1, 4, 10, 11, 20, 28, 37]
	*Prompt information provision*	[7, 13, 20, 36]
	*Participation in development*	[1, 11, 31, 36]
The importance of medical and organisational support	*Easier process*	[5, 18, 21, 28, 36]
	*As long as guidelines/policies followed, and information provided*	[15]
	*Organisational support and leadership are essential*	[1]
Midwifery champions	*Midwives promote and support water immersion*	[1, 4, 5, 11, 14, 15, 17, 19–23, 26, 28, 35, 36]
	*Champions are needed*	[4, 5, 10, 37]
	*Midwives offer water immersion as an option*	[1, 3, 11, 15, 37]
Policies and guidelines are often risk averse and do not reflect women's experiences	*Focus on risk and safety*	[7–10, 15, 31]
	*Precludes high risk, only low risk*	[7, 8, 10, 20, 21, 31]
	*Inconsistencies in guidance and contraindications with little underpinning evidence*	[7, 8]
	*Authoritative, prescriptive, restrictive, did not include women's views*	[7–11, 14, 21, 23, 36, 37]
	*Not reflective of contemporaneous evidence*	[1, 7–10, 15, 16, 20–23]
	*Normalise intervention*	[23]
Resistance stems from fear, lack of experience and support and the view that labour and birth are inherently risky	*Obstetricians lack training and experience*	[10, 20, 21, 28]
	*No support from obstetricians and/or seniors*	[1, 5, 6, 8–10, 15, 18, 20, 21, 25, 28, 29, 35–37]
	*Legal and insurance barriers*	[1, 10]
	*Midwives’ resistance or lack of experience*	[21, 29, 37]
Infrastructure, cost, and concerns inhibit implementation and accessibility	*Resources, few or no pools or the room blocked*	[1, 5, 7–11, 14, 16, 19–22, 24, 29, 31, 37]
	*Maternal collapse and evacuation*	[11, 18, 20, 26, 29, 36, 37]
	*Culture*	[7, 20]
	*Staffing*	[5, 11, 28, 29, 37]
	*Midwives discomfort*	[14, 19, 20, 28, 36, 37]
	*Paperwork*	[23]
	*Cost*	[1, 21, 27, 29]
	*Safety of the baby e.g., drowning*	[26, 28, 35, 37]
	*Personal concerns*	[32]
	*Waterproof CTG*	[20, 21]
Women must actively seek and request water immersion	*Policies not woman-centred*	[8, 9, 11, 24]
	*No information antenatally*	[8, 11, 20, 21, 23, 29]
	*Women must ask*	[1, 8, 20–22, 24, 29, 37]
	*Midwives influence women’s access*	[1, 3, 8, 11, 20, 23, 25, 28, 29, 37]
	*If women don’t ask, there must be no demand*	[23, 29]
	*Not a primary option compared to other options*	[20, 23, 24]
The illusive experienced practitioner	*Option removed because experienced/accredited practitioner not available*	[7, 8, 10, 12, 23]
	*Accreditation or extra training required*	[1, 8, 10, 12, 23]
	*'adequately' and 'appropriately' 'experienced', 'qualified', 'registered', responsible', 'competent', 'educated' practitioner*	[8, 12]

Five interpretative themes arose from the data analysis—see Table 4: *The paradox of prescriptiveness, The experienced but elusive practitioner, Advocacy and aversion, Trust or Trepidation* and *It’s your choice, but only if it is a choice.*

**Table 4:** Interpretative themes

Review finding	Theme
Policies and guidelines can be facilitative and prompt implementation of birth pool use Policies and guidelines are often risk averse and do not reflect women's experiences	The paradox of prescriptiveness
Education, training, mentorship, and experience leads to knowledge, competence, and confidence in facilitating birth pool use The elusive experienced practitioner	The experienced but elusive practitioner
Water immersion is a midwifery option in demand that facilitates physical and psychological benefits and physiological birth Resistance stems from fear, lack of experience and support and the view that labour and birth are inherently risky	Trust or trepidation
Midwifery champions The importance of medical and organisational support	Advocacy and tensions
Women must actively seek and request water immersion Infrastructure, cost, and concerns inhibit implementation and accessibility	It’s your choice, but only if it is a choice

### The paradox of prescriptiveness

Policy and guidelines generated conflicting accounts across 21 studies, thus, revealing the paradoxical landscape of maternity care settings that was either highly facilitative of birthing pool use, or restrictive. In seven studies, policies and guidelines were discussed as a facilitator of birth pool use [13, 35, 36, 42–45]. While policies were often labelled as prescriptive, and guidelines less so, the existence of a document that guided or informed the practice of water immersion was often viewed positively [13, 36, 42, 45]. For midwives, a policy or guideline offered a level of protection and safety defining what was both achievable and acceptable within their context of practice. These parameters positively influenced the likelihood of medical and obstetric colleagues supporting water immersion [17, 42, 45–48]. In some situations, policies led to improved staffing levels which enabled birth pool use [42]. Conversely, the prescriptiveness of policy, while often limiting women’s accessibility to water immersion, was also viewed as a ‘necessary evil’ to ensure the option was available at all:

*The existence of a policy 'legitimises' waterbirth but could be interpreted as controlling and restrictive *[38].

Several midwives reported policies and guidelines assisted their decision-making and reduced their fear of working outside their scope of practice or the parameters of their workplace [34, 46]. In turn, this assured guideline-developers that midwives would be accountable should they not be working in line with the guidance [17]*.* Moreover, in some studies, policies and guidelines were valued as tools with clearly documented benefits and risks that could be shared with birthing women or people to facilitate informed decision making [45, 48]. Additionally, they informed risk assessments assisting midwives to identify potential deviations from normal and as such, deliver appropriate and timely action [45, 48]. This was found to promote practitioner confidence and where confidence improved, practitioners were more likely to facilitate water immersion:

*"I wanted to make sure that what we did was robust and would stand up and actually support midwifery practice for birth through water. So I wanted to have a strong educational framework to support it. Because ... I’m sure eventually there would be some kind of adverse outcome and we need to be able to show that we’ve got some rigor behind what we’re doing and why we’re doing it"* Clinical Midwifery Consultant [34].

Conversely, multiple studies reported policies and guidelines restricted women and midwives’ autonomy [13, 17, 19, 35, 37, 38, 48]. For example, the information was either too scant or overly cumbersome, inhibiting greater uptake of the option [19, 38, 39, 48]. Others reported that policies and guidelines were excessively risk-focused and that much of the content was focused on minimizing “potential” or “possible” risks, and rather than supporting individualized care for each woman [17, 19, 35, 37–39, 44, 48, 49]. Where blanket statements about risk or medical history were included as contraindications [19], midwives interpreted this to be a major barrier whereby the slightest deviation often precluded women from choosing water immersion [17, 35–37, 48, 49]:

*"What it tends to end up as if you’re low risk you can use the pool, if you’re high risk you tend not to be able to, even if you would be suitable … any woman that ends up on labour ward tends not to end up in a pool, and in the birth centre it would be routine"* Obstetrician [36].

### The experienced but elusive practitioner

This theme exemplified a common requirement of practitioners to undertake additional education or training to facilitate birth pool use while also exposing the barriers to becoming experienced enough to do so. In some cases, excessive demands on midwives to achieve waterbirth competencies (that were not required to facilitate pharmacological pain relief) and to become ‘experienced’- loosely defined, was a significant barrier to providing birthing pools as an option. For example, multiple studies highlighted that water immersion was not always included in midwifery programs and therefore, some midwives sought out water immersion education packages or courses to upskill [13, 34, 44, 45, 48]. While some midwives felt formal education was important [44, 45], others believed it was simply physiological birth and therefore within the scope of a midwife [15, 42, 45]; as such, extensive and prescriptive accreditation requirements or credentialing were not required [34]. Midwives in the study by Woodward [45] suggested that these additional training requirements might deter midwives from facilitating and/or discussing water immersion with women.

“…training should not be seen as ‘*any big deal as that will put midwives off waterbirth if it is thought to be so different*.’ Midwife [45].

Furthermore, two studies [36, 45] discussed that water immersion training and education was not given the same weight as other procedures. Midwives resented a lack of training during their education once they experienced the benefits water immersion afforded women [42, 45]. This was exacerbated where women sought water immersion but were denied access because an experienced practitioner was not available [19, 34, 38]. The need for accreditation or formal training, particularly in Australia, was identified as a major barrier [19, 34, 35, 39, 44].

*"All women have the opportunity to have water immersion in labour however staff in birth suite are not all accredited for water births hence water immersion is not used as freely due to the "risk" of unplanned water births, hence all staff need to be accredited with water immersion/water birth as the two go hand in hand.”* Midwife [35].

In one study, the policies and guidelines specified a set number of water immersion competency requirements [48]. This was also detailed in other studies however, these requirements were not based on high quality evidence as reflected by the inconsistencies from venue to venue [19, 34]. In some cases, midwives who had been facilitating water immersion for decades were expected to undertake training packages when moving from one hospital to another in order to prove their competency [13, 34]. Therefore, policies and guidelines could inhibit birthing women or people’s access to birthing pools through creating elusive expectations of midwives, notably not required for pharmacological pain relief methods.

Additional education and/or training, while not always viewed by midwives as essential, was identified as a facilitator of water immersion [17, 34, 42, 47, 50]. The literature revealed that midwives and other health care providers involved in caring for women using water not only felt more confident after engaging in workshops and study days but were also more likely to promote and advocate for its use [42, 47, 51]. This was particularly the case for emergencies including evacuating a woman from the bath/pool in the event they collapsed [48].

Midwives wishing to support women in the pool raised the importance of having an experienced midwife mentor to facilitate confidence and competence [36, 42–45]. An experienced mentor provided reassurance and safety, especially for those who were just starting out as they could explore their feelings and fear and gain vital skills [45]. Confidence and competence grew with each positive water immersion experience, building experiential evidence to counteract negative views held by other clinicians and counteract opinions based on cultural influences [17, 35, 43]:

*"Coming across to a unit that [is] quite pro waterbirth it was just a confidence building thing for me and having had a good eight years I would say now of regular exposure to waterbirths, that’s really helped my confidence"* Community Midwife [36].

Clinicians who were experienced, competent and confident were more likely to discuss water immersion with women and facilitate access [35, 43, 45]. However, prior experience and contextual factors were found to be highly influential over the accessibility of water immersion.

*"When midwives are really confident in high risk … their high-risk care, starts to drip into the midwifery-led [unit], transfer rates go up, intervention rates start to go up. Whereas if you see it the other way, their normal care starts to get infiltrated into the women [on the obstetric unit]. So you see a peak in the pool being used [on the obstetric unit], because it’s a midwife that’s really confident with waterbirth"* Midwife [36].

Conversely, midwives who were mentored by practitioners (including midwives) averse to water immersion expressed a negative experience and a lack of confidence even where they wished to offer the option to women [20, 37, 45].

### Trust or trepidation

There was a generalized trust in water immersion from midwives included in the studies reviewed, especially from those who had facilitated the option. Water immersion was viewed as a means of supporting women and birthing people to achieve a physiological birth such that it had been associated with reduced intervention and improved outcomes [13, 19, 42, 51–54]. This view was informed by the wider research and the midwives’ own experiences of witnessing the comfort, protection and relaxation that birth pool use afforded women [13, 17, 50, 53, 54]. Midwives also discussed a deep trust in labour and birth as physiological processes and more so, in women’s bodies to achieve and navigate the intricate but intimate experience [52]. They articulated the demarcation of the birth pool instilled control in, and empowered the woman to work with their body to birth without interference [17, 51]:

*"You absolutely see the hormones that promote labour take over. You know labour progresses better and the woman relaxes into labour."* Midwife [51].

Conversely, obstetricians and other medical professionals in the studies were reported as unlikely to have been trained in, or to have witnessed labour and/or birth in water—this was associated with fear or misunderstanding [36, 37, 50, 55–57]. In some situations, this manifested as “fear mongering”, sabotage, threats, or other obstructive mechanisms [36, 56]. Some information leaflets included explicit statements suggesting that doctors “will not support water births” [50, 56]:

*"Our doctors are the stumbling block regarding birth in water. We do have support for labour in water. If we have an inadvertent water birth an [incident report] has to be completed and follow up is usually punitive despite the fact that there have been no negative outcomes and a high level of satisfaction from clients- they have actually refused to get out of the bath and have progressed to wonderful births. My own compromise at the moment is to have women get out of the bath late and continue water by means of the shower. Clients have an understanding of the lack of medical support and are ok with this compromise. Seems a ridiculous opposition to something that is actually an endorsed protocol for our unit."* Midwife [35].

Some midwives expected opposition from their medical colleagues [17, 45]. They put this down to ignorance and lack of understanding around the practice of water immersion and selective use and interpretations of the evidence base [17, 35, 56]. Midwives perceived that medical professionals primarily focused on the safety of the baby and argued the commonly held medical view that there remained insufficient high quality, empirical evidence to support water immersion and especially, for birth [19, 35, 37, 45, 47, 50, 56]. This was noted in the greater support for water immersion during labour compared to birthing in water [15, 37, 47] and was further supported by studies that suggested medical professionals and especially, paediatricians and neonatologists commonly challenged the notion that water immersion was a natural way to give birth [35, 37, 57]:

*"I think the idea of waterbirth is mis-sold to women [as] a physiological way to deliver babies. When actually the only mammal that deliver under water are whales, and even they don’t actually deliver under water…all the whales circle round and create a sort of bubble raft in order to make it more safe"* Neonatologist [37].

Newnham et al. [38] explored the way this aversion translated to policies, guidelines and associated information leaflets and compared these with the equivalent for epidural use. Their findings highlighted that epidural was much more acceptable and readily promoted. They argued that epidural constituted “acceptable risk” while water immersion was a step too far, highlighting pharmacological methods of pain relief were more readily supported and available when compared to water immersion [19, 38]. Thus, reflecting the contradictory nature of the risk averse culture of maternity care [35, 38, 39].

In the defense of medical professionals, midwives related their aversion to a lack of experience and engagement with water immersion [35–37]:

*"… some of the senior staff's thinking… Just things like water births. There's a lot of fear around the staff who have never seen a baby born in water or who just trust in the process of normal birth… I feel for those people and for the doctors as well because … you only see them when things are going wrong so I understand it's very difficult for them to trust in the process when they only ever see the process go wrong… they become very obstructive about making that happen… I think maybe there's a lot of ego stuff that goes on as well…”* Junior Midwife [52].

Some midwives discussed how they had held similar negative views about the practice before they have witnessed women using water [37]. However, negative views were not unique to medical professionals with some midwives still expressing uneasiness about water immersion [37, 45, 52]. Where these midwives held senior roles, this often inhibited access and therefore women’s choice [37, 52]. Therefore, birthing pool use was influenced by the prevailing care culture.

### Advocacy and tensions

This theme reflects the opposing views and perceptions of water immersion and how this manifested in environments that were either facilitative or inhibitive of birthing pool uptake. Midwives looked to midwifery champions for support and guidance to overcome their personal fears and concerns and to advocate for continued access and protection of the use of birth pools [36, 42, 45]. One midwife in Bayes, Juggins, Whitehead and De Leo’s [56] study stated, *"if you don’t have …a leader with vision, then you don’t have anything."* Water immersion champions were the most important enabler and their presence and persistent advocacy ensured that challenges and barriers were not only managed but kept to a minimum [35, 44, 45, 50]. These leaders were described as good communicators—they could articulate the benefits while mitigating against risks [45]. They were well versed and well-read and utilized evidence to gain the support of their medical counterparts.

The opposite was also true. In the absence of a midwifery water immersion champion or leader, conflicts between midwives and their managers could occur [45]. With a lack of leadership or ‘champion’ midwives were left to navigate restrictive birthing pool policies/guidelines individually—using “subtle and covert” mechanisms to navigate the challenges and barriers when supporting women’s informed choices [35, 38]. In some situations, this extended to “deliberate, accidental water births” where midwives would intentionally not ask the woman to leave the bath despite their local policy stating that water births were not supported [17]. Such behaviours risk furthering tensions and conflicts between maternity professionals and the endangering the water immersion service.

While midwifery champions were essential to the success of water immersion implementation, the lack of support from medical colleagues was often a “stumbling block” [15, 35, 36, 50]. Even where obstetricians, neonatologists and pediatricians supported water immersion for labour, there was a general view that waterbirth was associated with greater risk than traditional births [37, 50]. Midwives commonly felt that their clinical decision making and judgement with respect to the woman’s suitability were diminished and as such, their autonomy constrained due to medical aversion within their setting [19, 35]. A doula in Milosevic, Channon, Hughes, Hunter, Nolan, Milton and Sanders’ [36] study discussed that this was often dependent on the doctor or consultant:

*“It varies from consultant to consultant as to how woman centred they’re prepared to be. So you might find that somebody will agree something in advance… and then the consultant on the day is just not comfortable with it, the risk will have always been the same. What changes is the consultant who is there”* Doula [36].

Midwives described the lack of support from medical professionals as burdensome in that if anything were to go wrong, they would be held personally responsible. They referred to this as being “on your own” and “if anything goes wrong, it’s on your shoulders” [37].

### It’s your choice, but only if it is a choice

Thirteen studies highlighted the conflict/paradoxical positioning of ‘choice’ for women seeking to use a birth pool. In two studies, antenatal discussions around water immersion were limited and here, women had to proactively seek out information as it was not readily offered or discussed [19, 36]. Newnham, McKellar and Pincombe [39] reported that for women requesting to use the water, consent forms were required (in some cases, more than one) to be signed antenatally. While midwives mentioned that this prompted staff to talk about water immersion, they also described that this offered false reassurance as access to the pool/bath could not be guaranteed:

*"Three rooms have a bath…but you must have a signed consent form, have done all the paperwork before you come in, and not only do you need to meet all of the criteria, but there needs to be midwife on who is accredited, and there needs to be a bath free"* Midwife [39].

Moreover, even where a pool or bath was in place, infrastructural issues including access to a hot water supply, lifting equipment for evacuation, non-slip floors, a plug (in the case of fixed pools) and appropriate means of draining pools (for portable versions) were inhibitory [13, 44, 49, 51, 58]. In some cases, plugs were not available or were locked away, rendering baths useless and women or birthing people unable to access water immersion [35, 49]. Where infrastructural issues were overcome, other challenges were common. This included limited access to a suitably credentialed or qualified midwife—either they were not available, or staffing did not permit [19, 34, 45, 50, 56]. Where policies and guidelines required a midwife to be with women at all times while in the pool [45, 48], this could not be facilitated [20, 45]. In the absence of an experienced or waterbirth accredited midwife, women were precluded from accessing water immersion [56]. These issues often inhibited water use even before women were assessed or risked-out against prescriptive and rigid inclusion and exclusion criteria.

*"If the ward’s busy, they know that if that midwife goes in that room, (pool room) they’ve lost her... She doesn’t come out again, so that’s taken a member of staff away, whereas if we’ve got somebody on a bed with an epidural and a CTG (fetal monitor), you can come out occasionally and admit somebody else."* Midwife [20].

Additional issues were also described. In some units, limited resources (i.e., more than one birth pool, sufficient hot water, etc.) often denied women the option [20, 36, 37, 45].

*"There’s one pool in the whole [unit] and it is first come first served... I think I had that in my head …just even if I asked for it I probably wouldn’t get it"* Woman [36].

Midwives also described that the pools (particularly those that were fixed) were not conducive to their comfort and limited access to the woman and baby where indicated [17, 45, 50, 58].

*"Because of the height of the bath I can't sit down because I can't see. So I'm standing, leaning on my knees as a lever on the bath, leaning over, holding the torch with my left hand, holding the mirror with my right – it hurts. I've got a really sore back."* Midwife [58].

In a minority of cases, water immersion was seen as a costly option in terms of the initial outlay of installed birthing pools [37, 59] but were seen as cost-effective over the longer term. For example, Pagano et al. [59] identified it was a cost-effective intervention due to the reduced perineal trauma from water immersion.

## Discussion

This mixed methods systematic review included 37 papers to examine the facilitators and barriers of water immersion for labour and birth from the perspectives of multi-health professionals (midwives, obstetricians, neonatologists, maternity support workers, doulas etc.), revealing a complex interplay of factors. When personal, contextual, and infrastructural factors are aligned and directed towards the support and facilitation of birth pool use, water immersion is made a viable option. On the contrary, the more barriers that women and midwives experienced, the less likely water immersion was an option, reducing women and birthing people’s choices and access to a safe form of pain relief that is associated with excellent maternal-neonatal health outcomes. The barriers were multifaceted but weighted by policies and guidelines that were prescriptive and restrictive. These conveyed strict exclusion criteria for those who could access a birthing pool, but with little to no underpinning evidence and were unduly risk focused, starkly different to those policies/guidelines written for pharmacological pain relief options. These issues were reflected in a systematic review of scholarly references exploring publications authored by multi-professionals that focused on water immersion for childbirth [60]. For example, authors of obstetric or neonatology water immersion publications were less likely to reference midwives and nurses research in this area, and more, those from the latter were more likely to cite commentaries and case studies, rather than primary research with robust research methodologies. Accordingly, the authors found barriers to the diffusion of midwifery or nursing water immersion research into obstetrics or neonatology [60], which may subsequently limit the composition of evidence that inform water policies/guidelines as we have found in this review.

Compounding this, our review revealed that there was a need for a suitably ‘qualified’ and experienced clinician to be available and present at the birth. Water immersion was often eliminated as an option where this requirement could not be fulfilled. However, this negates the expertise and role of professional midwives in facilitating physiological land births, whereby these skills can be transferred to care in birthing pools. Many studies in our review identified a lack of exposure to water immersion for labour and birth so midwives’ skillsets were underutilised and magnified concerns toward a more risk-focused approach resulting in less, or no birthing pool provision. Our review revealed the challenges that midwives they face in implementing, facilitating, and advocating for this option especially when compared with pharmacological forms of pain relief [1]. Even where these barriers were not inhibitive, infrastructural issues including suitable bath/pools and access to a sufficient hot water supply meant that women could not access water even where they were deemed suitable. These insights provide important findings as to why women ‘struggle to get into a pool room’ [13–15, 40] which is counter to what many women or birthing people want. For example, a systematic meta-thematic synthesis explored women’s experiences of water immersion revealed benefits included analgesic properties and beyond—to an improved sense of control and empowerment and an easier transition from labour through birth to the postnatal period [8]. Such qualitative insights are strengthened by the maternal health benefits recently captured within a meta-analysis of 157,000 mother-baby dyads [5] (discussed in the background)—therefore, it is unsurprising that there is an increased demand for birthing pool use during childbirth [9]. Therefore, it is essential that the barriers to birthing pool use that we have identified in this review are overcome.

The findings of this review offer insight into the paradoxical reality of water immersion with each of the five themes detailing how the “swing” within these factors directly affected whether water immersion was facilitated or inhibited. These themes also provide insight into the work that still needs to be done to ensure that access to water immersion is improved, especially considering the growing evidence base that reflects that water immersion outcomes are equivalent if not, better, than those achieved in traditional births. Policies and guidelines must be informed by the most up to date evidence and evidence reviews must be complete and appropriately translated to these documents. For example, our review also found, converse to the barriers identified, policies and/or guidelines could also be facilitative of birthing pool use, women’s choice and midwives’ ability to deliver such care. As was the support of medical professionals towards birthing pool use and birthing women or people’s bodily autonomy. Where the culture supported water immersion as a viable option, and was driven by a midwifery champion, barriers were removed, and water immersion embraced by the broader multidisciplinary team—pools were sourced, and midwives and medical professionals advertised and discussed water immersion as valid, safe, and effective mode of birth. Thus, revealing insights as to how barriers could be overcome. Future research and clinical quality improvement projects should focus on exploring effective methods to ‘swing’ organisational cultures from inhibiting birthing pool use to a facilitatory approach. Additionally, such activities must include birthing women and people as part of the multidisciplinary team—those who have used birthing pools and those who wanted to but were denied. In this way, clinical practice and organisational cultural changes embed the principles of woman and person-centred care [61].

### Strengths and limitations

Strengths of this study included the use of a mixed methods evidence synthesis to source, collate and synthesize the literature using a systematic approach and we minimised bias through ongoing reflexivity and returning to the literature base to inform our interpretations. The inclusion of survey data. guideline analyses and an economic evaluation, which are often excluded from systematic reviews, strengthened this work and the overall findings. Overall, the strength of this approach meant we included 37 papers with a sample of > 9000 health professionals, from 11 high-income countries and one lower-middle income country, therefore, these findings have transferability to other high-income settings. However, as with all search strategies, there is a risk of missing pertinent studies. We mitigated against this through pilot searching, updating the searches at two time points, included citation chasing and cross referenced against Google Scholar. Additionally, with any interpretative syntheses there is a risk of under or over interpretation. However, we mitigated against this through developing descriptive statements in the first instance, which remained close to the original data, before embarking on an interpretative synthesis.

## Conclusion

This mixed methods evidence synthesis has explored the facilitators and barriers of birth pool use from organisational and multi-professional professional perspectives. Our findings revealed the paradoxical nature of water immersion as a practice. While water immersion is best facilitated in clinical contexts where multidisciplinary support is high and infrastructure is appropriate and available, the existence of a facilitative policy or guideline was also viewed as essential. Through a comprehensive search strategy and thorough mapping of the current evidence base, we have revealed the key facilitators and barriers to birth pool use. As such, the findings will be beneficial for directing further research and clinical quality improvement projects to implement and facilitate water immersion for childbirth.

### Supplementary Information

**Additional file 1.** Searches and Data Extract.

**Additional file 2.** QA.

## Data Availability

Included as Additional files.
